# COVID-19’s Impact on Burden and Nutrition for Family Caregivers of People With Parkinson’s Disease

**DOI:** 10.1093/geroni/igab046.1245

**Published:** 2021-12-17

**Authors:** Dara LoBuono, Rachel Robin, Mehmet Uygur

**Affiliations:** 1 University of Rhode Island, North Haledon, New Jersey, United States; 2 Rowan University, Glassboro, New Jersey, United States

## Abstract

The COVID-19 pandemic has worsened Parkinson’s disease (PD) symptoms; however how COVID-19 has impacted family caregivers of people with PD (PwPD) is unknown. A 38-item open-and closed-ended online survey that explored caregiver burden and nutrition behaviors during COVID-19 was completed by 34 caregivers. Quantitative variables related to how COVID-19 has impacted caregiver burden are reported as percentages. Responses to open-ended questions related to COVID-19’s impact on caregiver burden and dietary behaviors were double-coded by two researchers, differences in codes were discussed until consensus was reached, and themes were finalized. The mean age of caregivers was 67.2±8.7 (47-82 years of age) and the majority were female (64.7%). Since the COVID-19 pandemic, 61.7% of caregivers felt their relationship with their PwPD stayed the same or slightly improved, 41% reported having to make a slight or increased number of adjustments to their schedules to provide care and experienced a slight or increased physical strain because of providing care. 58.8% reported a slight or increased number of times they felt sad/hopeless and 76.5% reported a slight or increased number of times they felt anxious/worried. Themes related to COVID-19’s impacts on caregiver burden included: fear, stress, and isolation; increased caregiver responsibilities; no change in caregiving. Themes highlighting COVID-19’s impact on dietary behaviors included: healthier dietary patterns; increase in snack foods and boredom eating; no change in dietary patterns. Results suggest COVID-19 has negatively impacted caregiver well-being and further exploration in changes in dietary intake are warranted.

